# Interaction between Autophagy and Senescence in Pancreatic Beta Cells

**DOI:** 10.3390/biology12091205

**Published:** 2023-09-04

**Authors:** Francesko Hela, Cristina Aguayo-Mazzucato

**Affiliations:** Section on Islet Cell Biology and Regenerative Medicine, Joslin Diabetes Center, Harvard Medical School, Boston, MA 02215, USA

**Keywords:** cellular senescence, macroautophagy, diabetes, beta cells, SASP, senolytics

## Abstract

**Simple Summary:**

Cellular senescence is a state of replication arrest in response to different stimuli and has a wide range of effects on pancreatic beta cells. Accumulation of senescent cells impairs beta cell function and worsens the prognosis of diabetes. Macroautophagy is a lysosomal degradation pathway with the primary role of maintaining cellular homeostasis by clearing stress-inducing factors. It plays a context-dependent role in beta cells and the pathophysiology of diabetes. Even though these two processes converge in different signaling checkpoints our understanding of their relationship remains inconclusive. Filling this gap will enable us to grasp the full extent to which these pathways interact, leading to a better understanding of beta cell biology and diabetes. Addressing the questions related to these stress-induced mechanisms may open new research pathways in preventing and slowing diabetes and ameliorating beta cell functionality and health by means of novel therapeutic agents.

**Abstract:**

Aging leads to an increase in cellular stress due to the fragility of the organism and the inability to cope with it. In this setting, there is a higher chance of developing different cardiometabolic diseases like diabetes. Cellular senescence and autophagy, both hallmarks of aging and stress-coping mechanisms, have gained increased attention for their role in the pathophysiology of diabetes. Studies show that impairing senescence dampens and even prevents diabetes while the role of autophagy is more contradictory, implying a context- and disease-stage-dependent effect. Reports show conflicting data about the effect of autophagy on senescence while the knowledge about this interaction in beta cells remains scarce. Elucidating this interaction between autophagy and senescence in pancreatic beta cells will lead to an identification of their respective roles and the extent of the effect each mechanism has on beta cells and open new horizons for developing novel therapeutic agents. To help illuminate this relationship we will review the latest findings of cellular senescence and autophagy with a special emphasis on pancreatic beta cells and diabetes.

## 1. Introduction

Autophagy and senescence are two cellular mechanisms activated in response to a wide array of stressors. Autophagy is a lysosomal pathway that degrades different cargo, like damaged organelles, unfolded proteins, and invading microorganisms, in order to preserve cellular metabolic function independent of internal and external disturbances [1]. Cellular senescence is a state of cell cycle arrest induced by DNA damage, oncogenic activation, inflammation, oxidative stress, and mitochondrial dysfunction, among others [2]. Some of the senescent cells evolve a senescence-associated secretory phenotype (SASP) that recruits the immune system to clear dysfunctional senescent cells since they are resistant to apoptosis [3]. An increase in age leads to an increase in the senescent cell population and dysfunctional autophagy rate in different metabolic settings [4,5,6]. Their participation in different settings of aging has led to both being considered hallmarks of aging [7].

Pancreatic β-cells are key players in the body’s insulin homeostasis and, when subjected to stress, can lose function and lead to type 1 and type 2 diabetes [4,8]. Several studies have shown that clearing senescent beta cells or enhancing autophagy in beta cells ameliorates and even prevents diabetes [4,8]. However, there is a lack of reports showing the relationship between autophagy and senescence in β-cells even though they are both crucial stress response mechanisms. This review aims to give an overview of the separate and combined roles of autophagy and senescence in β-cells. The literature was systemically reviewed on MEDLINE using the freely accessible PubMed website as a search engine for the terms: “autophagy”, “senescence”, and “pancreatic beta cell” with the following combinations: “autophagy AND beta cells”, “senescence AND beta cells”, “autophagy, senescence AND beta cells”. Mainly the results of studies from the last 10 years were included and discussed but earlier studies were also included if they represented key contributions to the field.

## 2. Autophagic Processes

The discovery of lysosomes opened the pathway of catabolic research in the cell [9]. Autophagy is among the cell’s most important protective catabolic processes, activated when the cell experiences different stressors that disturb homeostasis [10]. It is a very well-conserved mechanism first identified and studied in yeasts, with the most common trigger being the scarcity of different nutrients like glucose or amino acids but not limited to it [11]. For this reason, the basic building blocks of the specific materials that are being degraded are recycled back into the system for the cell to use as an energy source [1].

**Figure 1:**
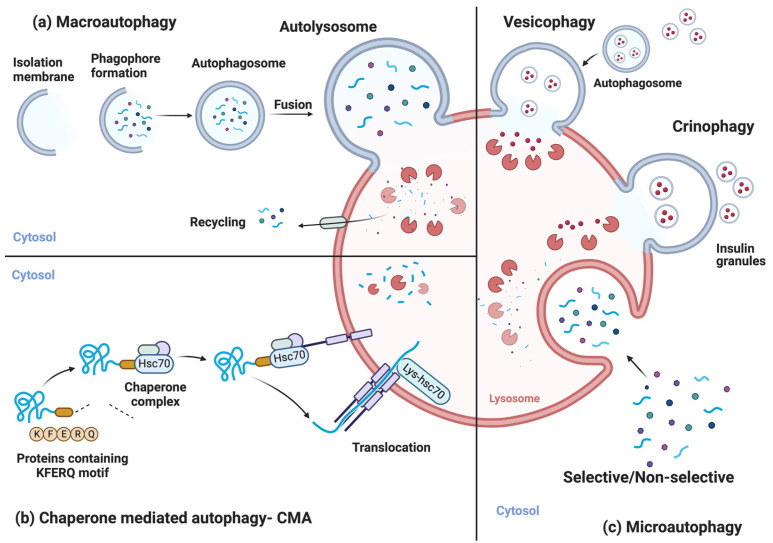
Overview of the autophagic processes in β-cells. (**a**) Macroautophagy, (**b**) chaperone-mediate autophagy, and (**c**) microautophagy are the three main autophagic processes that occur in β-cells. Crinophagy and vesicophagy of the insulin granules are the microautophagic mechanisms occurring only in β-cells. Figure made with BioRender.com.

Autophagy in the cell can be separated into three main processes. Macroautophagy (referred to as autophagy hereafter) is the most common type of autophagy the cell uses to adapt to disruptions to homeostasis. Targeted cargo like impaired organelles, intrusive bacteria, or cytoplasmic material is enclosed in a double-membranous vesicle named an autophagosome. Mature autophagosomes then fuse with the lysosomes, forming autolysosomes, in which cargo degradation is carried out with the help of the lysosomal enzymes. The constituents of the cargo material are recycled back to the cellular system for different uses [12] (Figure 1a).

Chaperone-mediated autophagy (CMA) is a particular form of autophagy, occurring only in mammals, explicitly targeting misfolded or damaged proteins containing the KFERQ motif. These proteins are first recognized by specific cytosolic chaperones and are degraded by the hydrolases and lysosomal enzymes after translocation to the lysosomes. The constituent amino acids are recycled back into the cell and used in protein synthesis and/or as an energy source [13] (Figure 1b).

Microautophagy is another crucial autophagic process that helps to maintain homeostasis in the cell and can be both selective and non-selective [14]. Different materials like organelles (peroxisomes (micropexophagy) and mitochondria (micromitophagy)), lipid droplets (microlipophagy), a subdomain of the ER (microER-phagy), a portion of the nucleus (micronucleophagy), and other cellular components can be degraded through microautophagy. Cargos are engulfed through membrane invaginations of the lysosomal membrane. With the help of lysosomal enzymes and hydrolases, they are degraded to be recycled back into the system [13]. Except for the selective microautophagy processes listed above, beta cells use two more specific microautophagic mechanisms. A specific form of microautophagy in β-cells is crinophagy which consists of the specific degradation of insulin granules by directly coalescing into the lysosomes [15]. This form of specific autophagy in β-cells is distinct from vesicophagy. During vesicophagy, insulin granules are first selectively taken up by the autophagosome and then directed to the lysosome for degradation [16] (Figure 1c).

Notably, all mechanisms of autophagy resemble each other in the pathway and induction stimuli that trigger them. These facts also explain the preserved mechanism from unicellular organisms like yeast to mammals [17]. Disabled autophagy is a hallmark of aging because it fulfills three conditions: (a) is manifested with age, (b) can accelerate aging when it is experimentally induced, and (c) aging phenotypes are reversed or inhibited [7]. This makes autophagy a valuable process at the intersection of many cellular processes.

## 3. Molecular Mechanism of Autophagy

**Figure 2:**
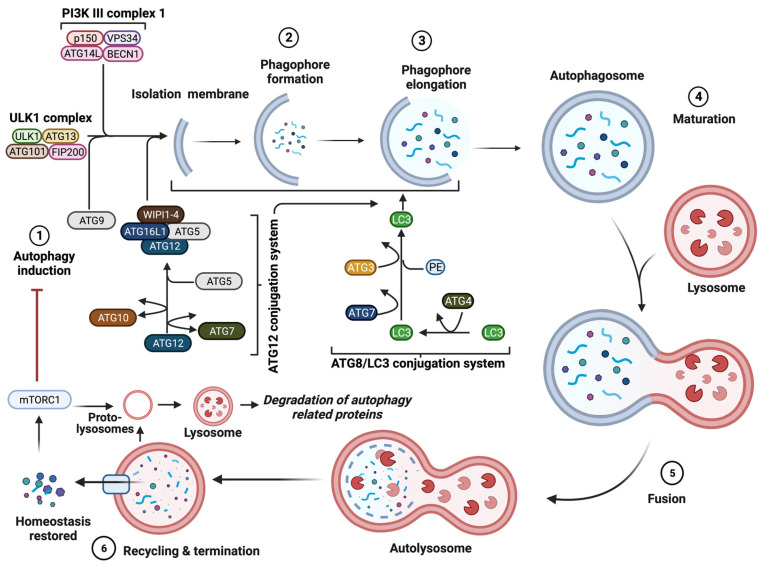
Autophagic molecular mechanism. (1) Induction starts with different signals triggering the signaling cascade, leading to the formation of the isolation membrane and later of the phagophore (2). Targeted cargo and further signaling make possible phagophore elongation (3). Sealing of the double-membranous vesicle forms the mature autophagosome (4). Transportation of the mature vesicle to the vicinity of the lysosome and fusion forms the autolysosome (5). Degraded cargo is recycled back into the system and the change in nutrition state signals the modulation of autophagy through mTORC1 (6). Figure made with BioRender.com.

The autophagic molecular mechanism can be categorized into five phases: induction and phagophore genesis, elongation, maturation, fusion, and termination. Sensing different cellular stressors directs several autophagic proteins to the specific cellular site where the isolation membrane will arise [18]. Isolation membrane components are thought to come from several sources like the plasma membrane [19], endoplasmic reticulum [18], and mitochondria [20]. Two complexes are vital in this initiation step, UNC51-like kinase 1 (ULK1) and class III PI3K complex 1 (PI3KC3-C1). During nutrient availability, the ULK1/2-ATG13-FIP200-ATG101 complex is bound to mTORC1, which inactivates ULK1 by phosphorylating it. Scarcity of nutrients inhibits mTORC1 phosphorylation, leading to ULK1 activation, which in turn phosphorylates the rest of the complex, causing induction of the autophagy [21,22]. The PI3KC3-C1 complex is composed of VPS34, BECN1, p150, and ATG14L. Interaction of this complex with phosphatidylinositol on the membranes produces PI3P [23]. Recruitment of other adaptor proteins like WIPI1-4, DFCP1, and different ATG proteins also makes possible interaction with the ULK1 complex [23]. All these interactions result in the synthesis of an isolation membrane, the autophagic vesicle’s source, and the downstream process (Figure 2).

Elongation of the isolation membrane is regulated by a pair of reactions triggered by ubiquitin-like ATG proteins. ATG12 is covalently conjugated to ATG5 and ATG16L1, employing ATG7 as an E1 enzyme and ATG10 as an E2 enzyme, forming the ATG12 ubiquitin-like covalent conjugation system [24]. The next step would be the conjugation of phosphatidylethanolamine (PE) to LC3-I by ATG7, ATG10, and ATG5-12-16L1 as an E1, E2, and E3 enzyme, respectively, forming the ATG8/LC3 ubiquitin-like covalent conjugation system [23]. This lipidated form of LC3 is a perfect marker of the autophagic flux since it is also an autophagic substrate [10]. Sufficient elongation of the isolation membrane leads to the sealing of the vesicle to form the double-membranous autophagosome. Clearing of all ATG proteins except LC3 and recruitment of different vesicle-SNAREs, target-SNAREs, and cytoskeletal motor proteins (kinesins) are among the markers of autophagosome maturation [25] (Figure 2).

Movement of the autophagosome towards the lysosome and the fusion step are regulated mainly by the Becn1-Vps34-Uvrag complex binding to Rubicon and EPG5 and INPP5E [26]. All these proteins aid in the tethering and fusion step by regulating SNAREs and cytoskeletal proteins. The final step, termination, is tightly regulated and keeps the rate of autophagy activation under control. Autophagy induction must be silenced when the cell’s homeostatic conditions are attained. This leads to mTORC1 activation, which triggers the synthesis of proto-lysosomal tubules. These tubules evolve into mature lysosomes with the primary function of degrading autophagy pathway components [27,28] (Figure 2).

## 4. Autophagy in β-Cell and Diabetes

The role of autophagy in the physiology of β-cells has long been studied but, lately, these studies have become more refined and systematic. It has been shown that the clearance of mitochondria from insulinoma cells is carried out by autophagy [29]. Also, when insulinoma cells are treated with liraglutide, a glucagon-like peptide-1 receptor agonist, an increase in the autophagy rate is observed. This GLP-1 receptor agonist also defends insulinoma cells from apoptosis, proposing a role for liraglutide in apoptosis by triggering autophagy [30]. Another GLP-1 receptor agonist, exendin-4, improves glucose tolerance by increasing insulin secretion in autophagy-defective β-cells. Surprisingly, there is no change in the level of autophagy, but the authors observed an increase in β-cell proliferation and a decrease in apoptosis [31]. In another study, data show that autophagy plays an important role in maintaining insulin granules in a balanced state when β-cells are experiencing defective insulin secretion [32].

Most studies using genetic manipulation techniques to target the role of autophagy in β-cells have been cell-specific since global knockout of autophagy genes, especially *Atg7*^−/−^, leads to neurodegeneration and death within 28 weeks after birth [33]. When the same gene was knocked out and β-cell-specific-Atg7-knockout mice were established, the authors observed an increase in apoptosis and a decrease in β-cell proliferation. This led to a reduction of secreted and serum insulin levels and β-cell mass. Organelle homeostasis in β-cells was also disturbed, as proven by endoplasmic reticulum distension, mitochondrial swelling, and piling up of ubiquitinated protein aggregates. Even though these autophagy-impaired mice experienced hypoinsulinemia and hyperglycemia, they did not become diabetic [34]. An active autophagic process plays an important role in the maintenance of β-cell function, mass, and structure, and any defects will cause, among others, hyperglycemia and insulin deficiency due to an abnormal function of cellular organelles. In another study, global *Atg7 (+/−)* haploinsufficient, heterozygote mice were generated. These mice showed no metabolic anomaly when fed a standard chow diet. When these mice were crossed with *ob/ob* mice or fed a high-fat diet, both of which introduce metabolic stress, diabetes was developed. Compared to *Atg7+/+-ob/ob* mice, autophagy-deficient ones showed aggravated insulin resistance, glucose intolerance, and increased lipid content [35]. These results indicate that an active autophagy process is needed to adapt to and protect against the induced metabolic stress affecting the pancreatic β-cells. This dynamic and adaptable, stimulus-induced autophagy is essential because basal autophagy is intact in mouse experiments when BCL2 is mutated in its phosphorylation sites using knockin techniques. These mutations lead to the prevention of stimulus-induced disruption of the BCL2–beclin-1 complex and impairment of autophagy activation. Still, there is no adaptability against a high-fat-diet-induced glucose intolerance even when the mice were exercised [36]. While mice constitutively expressing Beclin1, leading to continuously active autophagy, experience improved insulin sensitivity, they have impaired insulin secretion leading to glucose intolerance when fed on a high-fat diet [37].

In another study where a global overexpression of Atg5 was induced, an increase in autophagic activity, anti-aging phenotypes, and an extension of lifespan by approximately 17% were observed [38]. Caution should be exercised when extrapolating results from global or β-cell-specific autophagic gene deletions. When globally deleted, Parkin, a protein playing a role in mitophagy, impaired glucose tolerance [39]. At the same time, when specifically knocked out in β-cells, it exerted no change in β-cell formation, glucose tolerance, or insulin secretion [40]. These results suggest that an increase in adaptable autophagic activity would protect the cell against the induced systemic metabolic stress both at the systemic and the tissue level, as is the case of pancreatic β-cells.

β-cell autophagy also plays a vital role in diabetes with an essential emphasis on ER stress and unfolded protein response (UPR). This is supported by data showing that autophagy takes part in quality control of unfolded proteins and ER stress induced by obesity can be observed as an essential factor of β-cell failure in type 2 diabetes (T2D) [41,42]. These results were reinforced by data from Zucker diabetic fatty rats showing that autophagy plays a regulatory role in ubiquitinated protein aggregates that are formed and later degraded in β-cells in response to diabetes-induced oxidative stress [43]. Enhancing autophagy in Akita mice led to reduced ER stress due to proinsulin misfolding, milder diabetes, and lower apoptosis rate in β-cells [44]. When autophagy-deficient β-cells from specifically bred mice inducing ER stress by obesity were analyzed, a decrease in UPR gene expression and a more pronounced effect on the transition to severe diabetes were observed [45]. In the same study, autophagy-defective mice exhibited impaired β-cell function, increased apoptotic β-cells, and decreased β-cell mass compared to their counterparts [45]. When a more natural ER stress inducer, like palmitate, a free fatty acid, was used to treat INS-1, an increase in cell death was observed. This effect was reversed when autophagy was enhanced, while more aggravated cell death was seen when autophagy was blocked [46]. In the same type of cells and rat islets, enhancement of autophagy has a protective effect against hyperglycemia (glucotoxicity) and β-cell survival [47]. When systemic inducement of ER stress using an HFD was analyzed, severe glucose intolerance was observed in autophagy-deficient mice. This comes as a consequence of the mice being unable to increase the beta cell mass and maintain islet function to cope with insulin resistance caused by the HFD [48]. These studies show that UPR is not only a marker of ER stress but also an effector, with autophagy being a crucial mechanism employed by UPR in β-cells. The molecular mechanism behind this intricate relationship between these pathways is still unclear.

There are also contradicting reports from MIN6 cells and systemically from mice in which *Pdx1* was knocked down, showing that autophagy inhibition, not activation, improves cell death and survival rates. The same protective effect was also observed when autophagy-defective Pdx1-impaired mice were fed an HFD [49]. A study from T2D human islets showed that these islets have a higher autophagosome quantity than non-diabetic islets. When ER stress was induced in non-diabetic islets using free fatty acids, an increase in β-cell death and autophagic vacuoles was observed [50]. In another study, the same group showed that an increase in autophagy in T2D human islets reduced apoptosis, increased insulin secretion, and exhibited healthier mitochondria, ER, and insulin granules [51]. Taken together, these data show that different techniques of measuring autophagy activation through other markers of autophagic flux in specific steps of the process are needed to understand its physiological role in beta cells. At the same time, autophagy should be observed in detail at the cellular, tissue, and systemic levels with a significant emphasis on key signaling pathways that interconnect autophagy with other stress responses in the cell, such as senescence.

## 5. Cellular Senescence

The discovery that primary human cells have a finite number of cell divisions in vitro led to the definition of a crucial cellular stress response process: senescence [52]. In vivo, the same mechanism occurs due to exposure of DNA in the double-stranded chromosome end, leading to a DNA damage response through the ATM-p53-p21 axis [53]. A few of the different senescence inducers are chemotherapy, UV radiation, ER stress, and ROS load [54,55]. The cell uses senescence mainly as a tumor-suppressing mechanism that will halt the replication of a damaged DNA sequence, protecting against mutagenesis and further tumor progression [56]. At the same time, senescence has been observed to increase in many diseases like diabetes and during normal aging as a physiological process [57,58]. Senescent cells have a distinct phenotype with an upregulation of pro-survival mechanisms for inhibited apoptosis, bigger cell size, and increased activity of lysosomal hydrolase senescence-associated β-galactosidase (SA-β-Gal), and some, but not all, senescent cells develop a specific secretome, called the senescence-associated secretory phenotype or SASP [59,60,61]. SASP comprises an extensive array of factors like chemokines, pro-inflammatory interleukins, cytokines, and extracellular matrix-remodeling proteins, among others [60]. SASP plays a role in clearing out senescent cells because they are resistant to apoptosis. With an increase in chronological age, we observe both an impairment of the immune system effector cells and a higher induction of senescence, leading to an increased proportion of senescent cells in tissues [3]. Based on these data, senescence is considered a potential effector of aging phenotypes and many age-related diseases [62]. Based on different conclusions derived from other reports, there appear to be three types of senescence in vivo: acute, embryonic, and chronic. Acute senescence is activated in response to sporadic stressors required to maintain homeostasis in the cell, like in the process of wound healing [63]. Embryonic senescence is found throughout the embryo as a normal development process with an expression of p15^Ink4b^, p21^Cip1^, and different SASP factors [64]. Both acute and embryonic senescence processes seem to be favorable, and clearance of effectors is carried out in a timely fashion by senescence surveillance carried out by the cells of the immune system [65]. Chronic senescence, caused by increased cellular stress coupled with a failure to respond due to impairment of the molecular repair pathways, and senescence caused by exogenous stressors when curing different diseases are both disadvantageous processes [66].

Considering the vast number of cellular environments, triggers, and conditions in which senescent cells are formed in vivo, defining a global marker(s) for senescence has been cumbersome. Cell cycle inhibitors like p16^Ink4a^ and p21^Cip1^ are highly expressed and characteristic of senescent cells. However, they alone cannot be considered markers since different homeostatic cellular mechanisms or other conditions can lead to cell cycle inhibition [56]. Markers of DNA damage response like phosphorylation of the histone H2AX or p53 at the sites of DNA repair or senescence-associated heterochromatin foci are also characteristic of senescent cells but not unique to them [67]. SASP is also very diverse based on the type and length of the senescence process, trigger, and cell type [68]. Senescent cells also exhibit an irregular shape due to rearrangements of vimentin filaments and increased expression of caveolin-1 in the plasma membrane [69,70]. An increase in mitochondrial content and ROS synthesis with a decreased membrane potential and an increased unfolded protein response due to ER stress are also among the characteristics of senescent cells [71,72]. The absence of a pan-senescence marker(s) makes it mandatory to combine different characteristics of the senescent cells before being able to derive a conclusion on senescence status and progression.

Senescent cells are mainly produced in three primary settings during the human lifespan: biological aging, age-related diseases, and management of different diseases. Natural aging is a perfect example of the effect of physiological stresses like ER stress, DNA damage, telomere shortening, and increased ROS in the body, leading to chronic senescence [73,74]. These senescent cells have an increased SA-β-Gal activity and expression of cell cycle inhibitors. This phenotype is thought to be developed by the impairment of maintenance processes in the cell by SASP and the clearance of regenerative stem cells by senescence [75]. These conditions which increase with the increase in chronological aging make cellular senescence a hallmark of aging [7].

Senescence is also active during different disease conditions. Whether senescence is causative or arises due to disease states is still unclear in many disorders. In the pancreas, loss of proliferating β-cells due to senescence coupled with an increase in the inflammatory response from SASP activation in the adipose tissue can contribute to insulin resistance, glucose intolerance, and type 2 diabetes [76]. Since diabetes is an age-related disease, the role of senescence can be thought of as a two-step process. Initially, senescent cells pile up throughout life due to different molecular maintenance mechanisms employed to repair tissues. The second part begins when the organism is more fragile and less capable of regulating and clearing senescent cells, leading to their accumulation and tissue dysfunction.

## 6. Cellular Senescence in β-Cells and Diabetes

The proliferative capacity of β-cells to meet the demands of the increasing insulin resistance in the body leads to telomere shortening. This causes a decrease in these cells’ proliferative capacity, which can contribute to the development of T2D [77]. There is also a decrease in β-cell proliferation and responsiveness to glucose stimulation in old-aged mice and humans [78,79]. Under these conditions, there is an accumulation of senescent β-cells [80]. Using a senescent mouse model with a *Tert* knockout modification and short telomeres leads to increased glucose intolerance [76]. Reports from another study using a similar model showed an increase in p16^Ink4a^ in islets, a decrease in β-cell proliferation, and fasting hyperglycemia. There was also an impairment of mitochondrial membrane hyperpolarization coupled with Ca^2+^ in β-cells, both of which play a role in insulin exocytosis [81].

There exists an important relationship between inflammation in diabetes and SASP recruitment of immune effectors in β-cells. Studies show that macrophages are observed to infiltrate, accumulate, and polarize into pro-inflammatory type I in diabetic mouse models and islets of T2D patients [82,83]. B lymphocytes are increased while there is no change in T lymphocyte percentage in the islets of T2D mouse models [84]. High levels of free fatty acids (FFAs) are toxic to β-cells and, as a response, trigger the recruitment of pro-inflammatory macrophages to the islets. At the same time, inflammatory cytokines and chemokines are also secreted as a response to stress by FFA in β-cells. IL-1β is crucial since its secretion triggers the activation of resident immune cells. This response is mediated by the toll-like receptor 4-MyD88 pathway leading to NF-κB activation. Studies observed that inhibiting this pathway decreases IL-1β synthesis, enhancing insulin synthesis and secretion [85].

Our group has shown an increase in senescent cells in the islets of aged mice and humans, with a further rise in the islets of people with T2D. Insulin resistance and a high BMI also increase senescent cell presence in the islets [4]. An in vivo senescent cell reporter system employing the INK-ATTAC transgene, which specifically destroys the subpopulation of β-cells expressing p16^Ink4a^, has been a valuable tool for studying characteristic senescent β-cells. Clearance of this subpopulation of cells decreases the expression of senescence markers and SASP while improving glucose metabolism, β-cell function, and insulin secretion. Navitoclax, a pharmacological senolytic and potent Bcl-2 inhibitor, also improved β-cell gene profile of mice in a model of insulin resistance [4]. A senescent β-cell subpopulation from mouse islets was selected based on SA-β-Gal expression and RNAseq revealed that β-Gal^+^ cells had upregulated senescence genes (*p16^Ink4a^*, *p21^Cip1^*), SASP genes (*Ccl2*, *Il-1α*, *IL-6*, *TNF-α*)*,* aging markers (*Igf1r, Bambi*), and a decrease in expression of β-cell identity genes (*Ins1*, *Pdx1*, *Mafa*, *Neurod1*) [86]. The BCL-2 pathway, a critical anti-apoptotic mechanism, is also upregulated in SA-β-Gal+ senescent cells, consistent with the property of senescent cells being resistant to apoptosis. At the same time, Navitoclax clears them from the islet cells [4].

Senescent β-cells downregulate functional genes with roles in incretin signaling and insulin granule synthesis potentially leading to impaired insulin secretion [5]. We also showed that the knockdown of IGF1R in mouse islets upregulated β-cell identity and function genes downregulating senescence markers and improving glucose clearance and glucose-induced insulin secretion. These effects were maintained in β-IGF1R mice even when they were fed a high-fat diet. There was also an increase in autophagy and mTOR signaling pathways based on results from RNAseq analysis from islets isolated from β-Igf1rKD mice [87]. These data show a relationship between two hallmarks of aging, decreased nutrient sensing through the insulin-IGF1R pathway and cellular senescence in mouse β-cells, emphasizing the importance these hallmarks have in diabetes and the extent to which they affect each other [7,87].

An extended layer of complexity between senescence and autophagy in the β-cells is conveyed by the highly heterogeneous environment of the pancreas in which they reside. The pancreas has an endocrine and an exocrine portion, both of which play essential roles in maintaining homeostasis. The endocrine portion is composed of the islets of Langerhans which maintain blood glucose levels through insulin-secreting β-cells and glucagon-secreting α-cells. β-cells play a central role in the development of T2D and we have shown that adult humans (20–70 years old) have senescent β-cells [4]. In mice, senescent β-cells lose glucose responsiveness and secrete SASP factors [88]. Moreover, these senescent subpopulations are dynamic, and insulin resistance accelerates their appearance. Deletion of this senescent population (senolysis) improves β-cell function, increases expression of β-cell genes while decreasing expression of senescence and SASP genes, and lowers blood glucose levels.

The exocrine portion of the pancreas is composed of acinar cells and a ductal system. Pancreatic ductal adenocarcinoma (PDAC) has a very poor prognosis and is refractory to chemo- and immunotherapies. This is partly due to KRAS mutations that lead to hypovascularity, a pro-inflammatory environment, and immunosuppression. Another frequent inactivating mutation found in PDAC is CDKN2A/p16^Ink4a^, a classic marker of senescent cells. It has been shown that PDAC can be suppressed through induction of retinoblastoma-mediated senescence. This conversion induces the secretion of local SASP which includes pro-angiogenic factors that promote vascularization, enhancing drug delivery [89]. The exocrine pancreas is composed of acinar cells (>90%) which, under aging and stress, dedifferentiate and acquire an early pancreatic progenitor phenotype, concomitant with activation of the Ras-Mapk pathway and of a senescence program [90]. The dedifferentiated acinar cells were proliferation arrested by the acquisition of a senescent state, characterized by induction of p53, p21^Cip1^, p16^Ink4a^, and SA-β-Gal [91,92,93,94].

Similar to variation in senescence mechanisms in the different compartments of the pancreas, differences in autophagy have also been reported. For example, it has been shown that autophagy is essential to maintain acinar cell function to preserve the high rate of protein synthesis that characterizes the exocrine pancreas. Loss of autophagy can result in exocrine insufficiency leading to pancreatitis and might increase the risk of pancreatic cancer [95]. It has also been shown that the exocrine pancreas has a higher autophagy flux than the endocrine pancreas [96].

These studies highlight the heterogeneity in senescence and autophagy within the pancreas and underscore the importance of future studies addressing the potential influence of the specialized environment or metabolic factors on the interplay between autophagy and senescence.

## 7. Interaction between Autophagy and Senescence

**Figure 3:**
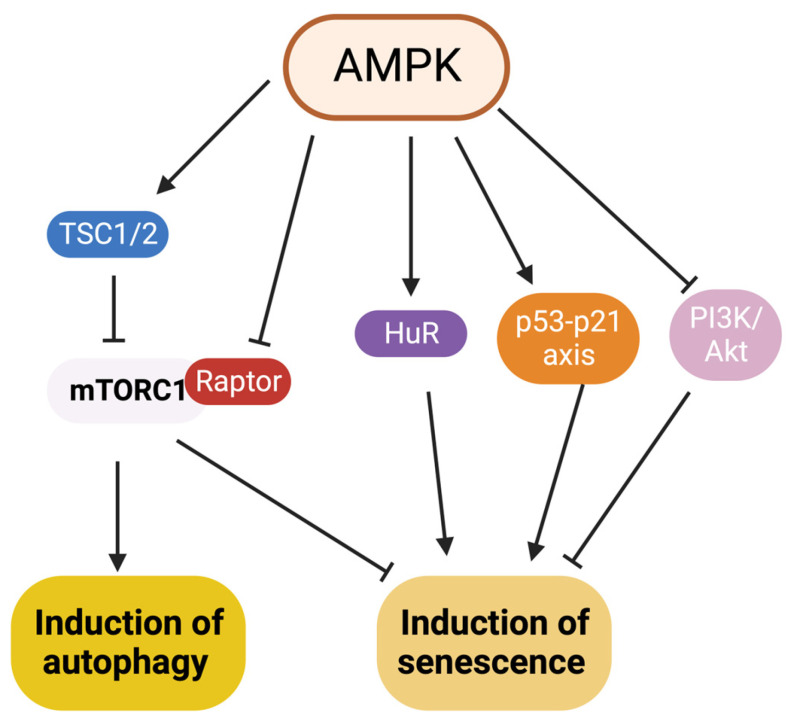
Role of AMPK signaling pathway in induction of autophagy and senescence. Figure made with BioRender.com.

Autophagy and senescence converge in inducing triggers and signaling pathways like the AMPK signaling pathway. The figure in this review is a simplified version of their mechanistic interaction and readers are encouraged to consult more specific reviews like the one by Wang et al. [97] (Figure 3). In terms of affecting each other, the relationship between autophagy and senescence is still contradictory [98]. Since the primary purpose of autophagy is to maintain cellular homeostasis by clearing stressors that cause senescence (e.g., removing damaged cellular components), it is expected to have an anti-senescent effect (Table 1). Still, some reports show autophagy leads to a pro-senescence role (Table 2).

It was observed that the action of autophagy inhibiting senescence maintained the stemness of the muscle stem cells in mice. Inhibiting autophagy in both biologically aged and young satellite cells activates senescence, increases oxidative stress and mitochondrial impairment, and decreases the quantity and function of satellite cells [99]. Restoring autophagy in geriatric satellite cells inhibits senescence and restores regenerative properties showing autophagy as a crucial muscle stem cell modulator [99]. Inhibiting autophagy by knocking down essential autophagy genes, ATG7 and ATG12, induces premature senescence associated with an increase in expression of senescence markers and ROS synthesis occurring in a p53-dependent pathway in human fibroblasts [100]. Pharmacologically inhibiting autophagy using bafilomycin A1, a vacuolar-type ATPase inhibitor inhibiting lysosomal degradation of autophagy cargo, impairs cellular proteostasis and induces premature senescence in the fibroblast MRC-5 cell line [101] (Table 1).

Autophagy also suppresses senescence under a “threshold” level of oxidative stress in the cell. When this stress level exceeds the autophagic response, autophagy is inhibited and senescence is induced [102]. Activating autophagy under this extreme oxidative stress condition by targeting key molecular targets like AMPK or mTOR inhibits senescence [102,103]. This scenario increases in complexity when three hallmarks of aging, disabled autophagy, cellular senescence, and mitochondrial dysfunction, intersect [7]. When mitochondrial dysfunction-associated senescence (miDAS) is activated, it inhibits growth and its associated SASP by activating the AMPK-p53 pathway [104]. Clearing impaired mitochondria by activating mitophagy leads to decreased expression of the primary senescence markers while increasing the glycolysis rate [105]. Another report showed that inhibiting ATM reduces senescence and its features mainly due to the action of autophagy [106]. These and other reports support an anti-senescence activity of autophagy in different tissues and cellular contexts [107,108,109] (Table 1).

However, there are reports supporting a pro-senescence effect of autophagy. They state that autophagy is activated during senescence and overexpression of an autophagic gene, ULK3, induces both autophagy and senescence. Impairment of autophagy, on the other hand, inhibits senescence and SASP secretion [110]. The same group also saw a connection with a specific type of autophagy, the TOR–autophagy spatial coupling compartment (TASCC), which induces the synthesis of some SASP proteins [111]. An increase in the expression of CDK inhibitors (senescence markers) enhances both autophagy and senescence in human fibroblasts and breast cancer cells [112]. Another study showed that inhibiting ATG5 in melanocytes leads to a bypass of the oncogene-induced senescence (OIS) in these cancerous cells, suggesting a pro-senescence role of autophagy [113]. Also, inhibiting autophagy inhibits lamin B1 degradation and decreases OIS in primary human cells [114]. Other studies also show a pro-senescence effect of autophagy on senescence in different tissues and cellular contexts [115,116,117,118,119] (Table 2).

**Table 1:** Studies on how autophagy inhibits/prevents senescence (anti-senescence role).

Autophagy Process	Type of Senescence	Cell Type	Ref.
Macroautophagy	Toxic cellular waste accumulation, resulting in entry into senescence	Primary murine muscle satellite cell	[99]
Macroautophagy	Premature senescence	Primary human diploid fibroblasts	[100]
Macroautophagy	Oxidative stress-induced senescence	NIH3T3 and MRC-5 cell lines	[102]
Macroautophagy	Oxidative stress-induced senescence	NIH3T, MRC-5 and HUVEC cell lines	[103]
Mitophagy	Mitochondrial dysfunction-associated senescence (MiDAS)	IMR90, BJ cell lines and Inguinal adipose tissue	[104]
Mitophagy	Oxidative stress-induced senescence	Human diploid and ataxia telangiectasia fibroblasts	[105]
p62-mediated selective macroautophagy	DNA damage response- induced senescence	IMR90 and BJ cell lines	[107]
Macroautophagy	Oxidative stress-induced senescence	SH-SY5Y cell line	[108]
Macroautophagy	Oncogene-induced senescence	MEF cell line	[109]

**Table 2:** Studies on how autophagy induces senescence (pro-senescence role).

Autophagy Process	Type of Senescence	Cell Type	Ref.
Macroautophagy	Oncogene-induced senescence	IMR90, BJ cell lines	[110]
mTOR-autophagy spatial coupling compartment	Oncogene-induced senescence	IMR90 cell line	[111]
Macroautophagy	Genetically inhibited senescence	hTERT-BJ1 and MDA-MB-231 cell lines	[112]
Macroautophagy	Oncogene-induced senescence	Melanoma cells and primary melanocytes	[113]
Macroautophagy	Oncogene-induced senescence	IMR90, BJ cell lines	[114]
Macroautophagy	Chemotherapy-induced senescence	MCF-7 and HTC-116 cell lines	[115]
Macroautophagy	Replicative senescence	MRC-5, WI-38 and p53-null immortalized fibroblast cell lines	[116,117]
Macroautophagy	Radiation-induced senescence	AMC-HN-9, U-87, U251, A549, HT-29, MDA-MB-231 cell lines	[118]
Macroautophagy	Radiation-induced senescence	MDA-MB-231 and PTTG1-knockdown MDA-MB-231-2A cell lines	[119]

Based on the current data, autophagy has a dual, context-dependent role in senescence. There might be different reasons behind this seemingly controversial effect. In the case of selective autophagy, the autophagy process that specifically degrades one substrate, the relationship between substrates and the receptor is thought to affect the role in senescence. LC3B-lamin-B1-dependent selective autophagy promotes senescence while the p62-GATA4-dependent selective autophagy inhibits it [107,114]. Identification and study of autophagy receptor–substrate pair(s) might unravel another layer of mechanistic control on senescence by autophagy. The time and which type of autophagy starts to act after the cell has been introduced to a stressor also play a role in this controversy. General autophagy inhibits senescence early in stress-induced signaling since its main role is to maintain cellular homeostasis. After a threshold of time during which the cell has lived under stressful conditions has been reached, general autophagy starts to promote senescence by sustaining the senescent cell population and ensuring its viability [120]. The part of the cell in which autophagy acts also might play a role in the effect on senescence especially in highly polarized cells like epithelial cells. TASCC and nuclear autophagy are two examples of this compartmentalization [111,114].

These studies evidence our incomplete knowledge of how these two critical processes (autophagy and senescence) interact in vivo, specifically in β-cells and in the setting of diabetes. Further studies on the mouse and human islets and their relationship with obesity, insulin resistance, and T2D are needed to shed light on their interaction since the only FDA-approved autophagy inhibitors are in the setting of cancer therapies [121].

## 8. Are Autophagy and Senescence a Potential Therapeutical Target in Diabetes?

Impairment of autophagy due to age, disease, or exogenous stress would dampen or inhibit the renewal of dysfunctional ER or mitochondria in β-cells, worsening the prognosis of T2D [122,123]. These results suggest that enhancing autophagy locally in β-cells, either pharmacologically or genetically, might have beneficial effects in preventing or treating diabetes. Some widely used diabetes drugs like metformin, GLP-1 receptor agonists, and rosiglitazone (a PPAR agonist) have been shown to partly exert their beneficial effects through enhancing autophagy. Both metformin and rosiglitazone have been reported to defend β-cells from lipotoxicity and increase autophagy through an AMPK-dependent pathway [124,125]. Chronic activation of AMPK can lead to detrimental effects for β-cells; also, metformin can work through multiple non-AMPK pathways [126,127]. Due to this, it is currently unclear what would be the rate of autophagy enhancement that would lead to the improvement of diabetes by the actions of metformin or rosiglitazone. Rapamycin, an autophagy activator inhibiting mTORC1, can also worsen the diagnosis by leading to an increase in insulin resistance and reducing β-cell mass and function, adding reasons for caution while considering autophagy activation as a therapeutic goal in diabetes [128]. Other autophagy-activating compounds like trehalose or imatinib have been considered as therapeutics [129]. When trehalose was administered to mice expressing human amyloidogenic IAPP, it enhanced islet autophagy and improved glucose profile and β-cell function coupled with a decrease in accumulated amyloid in islets [130]. Most of the studies mentioned above were conducted in artificial models using genetic knockout or overexpression. In contrast, more studies are required to check the effect of autophagy activation in mouse and human physiological models. Other details that should be taken into account when thinking of autophagy as a therapeutical goal are the cellular context, rate, severity, and duration because autophagy has different effects in different tissues and cellular settings [131].

Senescence can be induced in various chemotherapeutic treatments to lead to upregulation of pro-senescent p53 signaling or using cell cycle inhibitors to activate senescence in cancer patients [132,133]. A drawback of these strategies is the generation of a pool of tenacious senescent cells that secrete SASP effectors leading to detrimental side effects on the healthy surrounding tissue even after cancer treatment is finished. For this reason, there have been attempts to combine these pro-senescence therapies in the short term with therapeutics that would clear senescent cells in the long run. The most direct way of removing senescent cells is by induced cell death using senolytic compounds or by inhibiting their secretome, SASP, or other markers using senomorphic molecules. Since senescent cells use pro-survival pathways to avoid apoptosis, using inhibitors to these pathways is a logical strategy [134]. Dasatinib, a kinase inhibitor combined with quercetin, a plant flavonoid, shows senolytic activity in cultured cells, mice, and humans [135]. Different molecules like HSP90 inhibitors and Navitoclax are also used as senolytics, with more compounds coming and being FDA-approved [122,136,137]. Targeting SASP using senomorphics that target upstream signaling pathways like mTOR (rapamycin), NF-κB, p38MAPK, and JAK-STAT would also be an attractive opportunity [138]. At the same time, an already widely used anti-T2D drug, metformin, is also a senomorphic inhibiting NF-κB activation and decreasing SASP [139]. Taking this strategy into the clinic would be possible only when we can target precisely the senescent-associated pro-inflammatory factors of SASP and also discern in terms of exocytosis and secretion between the beneficial and the harmful SASP. A potential therapy would be to raise antibodies against senescent-specific antigens to direct cytotoxic T cells or engineered nanoparticles [140]. Another possibility would be to target those antigens by engineering T cells with chimeric-antigen receptors (CARs) [141]. A detail to be considered in pancreatic β-cells is the effect clearing senescent cells would have on beta cell mass. In the specific setting of beta cells, we recently showed that deletion of p16^Ink4a^ cells in a transgenic mouse model resulted in limited effects of proliferation and no changes in beta cell mass [142]. Targeting senescence is a promising strategy in many age-related diseases. However, a particular emphasis would need to be placed on eliminating side effects by killing only harmful senescent cells and accomplishing efficient clinical advantage. All these strategies can be transferred from bench to bedside after safety has been proved in all the settings where these compounds would be used.

## 9. Conclusions

The relationship between senescence and autophagy in β-cells and diabetes described in this review is intended to provide an overview of the complexity of these intertwined mechanisms. Developing therapies against diabetes has been focused more on senolytics which are not β-cell-specific. There is a similar situation in the evaluation of autophagy modulators. An important factor that needs to be taken into account is that autophagy promotes cancer cell survival and its inhibition aids the treatment of different cancers [143,144]. Since these modulators would be required to be administered over an extended period, caution should be exercised while preventing diabetes to not promote cell transformation and uncontrolled growth. In this setting, the importance of senescence for autophagy in diabetes and other diseases is underscored. More detailed studies are required to determine when and where different types of autophagy act during senescence and T2D progression. Being two hallmarks of aging with roles in diabetes, aging, neurodegeneration, and metabolic diseases, key nodes of interaction between them could lead to novel targets in the prevention and treatment of these diseases, having an ultimate positive effect on healthspan.

## Data Availability

Not applicable.
